# Semi-Automated Recording of Facial Sensitivity in Rat Demonstrates Antinociceptive Effects of the Anti-CGRP Antibody Fremanezumab

**DOI:** 10.3390/neurolint15020039

**Published:** 2023-04-29

**Authors:** Nicola Benedicter, Karl Messlinger, Birgit Vogler, Kimberly D. Mackenzie, Jennifer Stratton, Nadine Friedrich, Mária Dux

**Affiliations:** 1 Institute of Physiology and Pathophysiology, Friedrich-Alexander-University, D-91054 Erlangen, Germany; 2 Teva Pharmaceuticals, Redwood City, CA 94063, USA; 3 Department of Physiology, University of Szeged, H-6720 Szeged, Hungarydux.maria@med.u-szeged.hu (M.D.)

**Keywords:** fremanezumab, monoclonal antibody, behavioral model, mechanical sensitivity, thermal sensitivity, calcitonin gene-related peptide, migraine symptoms

## Abstract

Migraine pain is frequently accompanied by cranial hyperalgesia and allodynia. Calcitonin gene-related peptide (CGRP) is implicated in migraine pathophysiology but its role in facial hypersensitivity is not entirely clear. In this study, we investigated if the anti-CGRP monoclonal antibody fremanezumab, which is therapeutically used in chronic and episodic migraines, can modify facial sensitivity recorded by a semi-automatic system. Rats of both sexes primed to drink from a sweet source had to pass a noxious mechanical or heat barrier to reach the source. Under these experimental conditions, animals of all groups tended to drink longer and more when they had received a subcutaneous injection of 30 mg/kg fremanezumab compared to control animals injected with an isotype control antibody 12–13 days prior to testing, but this was significant only for females. In conclusion, anti-CGRP antibody, fremanezumab, reduces facial sensitivity to noxious mechanical and thermal stimulation for more than one week, especially in female rats. Anti-CGRP antibodies may reduce not only headache but also cranial sensitivity in migraineurs.

## 1. Introduction

Calcitonin gene-related peptide (CGRP) is a vasodilating neuropeptide released upon activation of nociceptive afferents in the trigeminovascular system [1]. CGRP is believed to play an important role in migraine pathopysiology, even if the pathomechanisms involved are not yet fully understood [2]. Infusion of CGRP can cause an immediate headache and it is able to trigger delayed migraine-like states in parts of migraineurs with and without aura [3,4]. Based on accumulated knowledge on CGRP, different classes of therapeutics against migraine targeting the CGRP pathway have been developed [5], among them humanised monoclonal anti-CGRP antibodies, which are effective in preventing frequent and chronic migraines [6]. In preclinical models of migraine, anti-CGRP antibodies such as fremanezumab prevented increased trigeminal activity following cortical spreading depression in rats as a likely correlate of migraine aura [7,8]. 

Facial mechanosensitivity has been determined in rodent models as a measure of facial hypersensitivity in migraine [9,10]. The involvement of CGRP in the control of facial sensitivity is not entirely clear. In mice, intrathecal application of CGRP can cause facial allodynia with elevated expression of the receptor activity-modifying protein-1 (RAMP1), the functionally rate-limiting subunit of the CGRP receptor [11,12]. Intraperitoneal injection of CGRP induced grimace responses which are indicative of pain [13], but the sensitivity to CGRP seems to depend on the mouse strain [14]. Injection of CGRP into the facial skin has been found to cause dose-dependent periorbital allodynia, which could be prevented by administration of the CGRP receptor antagonist olcegepant or a monoclonal antibody against CGRP [15]. In rats, injection of CGRP into the facial skin did not induce signs of facial pain unless the rats were sensitized by pre-administration of nitroglycerin [16]. Remarkably, the application of CGRP to the rat dura mater has been shown to cause periorbital hypersensitivity selectively in female animals [17]. Similar responses in different species to CGRP administration through different routes have been reviewed in detail [18]. Assuming that CGRP is involved in facial sensitivity, the hypothesis of the current study was that anti-CGRP antibodies may reduce facial sensitivity in rats. 

Using a new orofacial stimulation test device combined with feeding of an attractive solution, we measured facial sensitivity using different parameters such as the number of approaches to the source and the time of consumption before and after treatment with the anti-CGRP antibody fremanezumab or an isotype control antibody that does not target CGRP in rats of both sexes. In addition to a mechanical barrier that allows the approach to the source only under facial noxious mechanical stimulation, the tool also offers the option to apply a thermal barrier, which is a type of stimulation that has limited use so far regarding facial sensitivity. As a main result of the current study, both mechanical and heat barriers reduced the consumption parameters indicating unpleasant facial sensations that lead to avoidance of the barriers. Pre-treatment with fremanezumab partly abolished these changes, suggesting the involvement of CGRP in facial nociceptive sensitivity in rats. 

## 2. Materials and Methods

### 2.1. Animals

Female and male Wistar rats with a body weight between 200 and 460 g bred and housed in the animal facility of the Institute of Physiology and Pathophysiology were used. Animal housing and all experiments were carried out according to the German guidelines and regulations of the care and treatment of laboratory animals and the European Communities Council Directive of 24 November 1986 (86/609/EEC), amended 22 September 2010 (2010/63/EU). Groups of 3–4 animals were kept in cages in a 12 h day–night-cycle and received pellet food and water ad libitum. Equal numbers of animals were matched according to their sex and weight and allocated to the treatments. The estrus state of females was not checked. The experiments were performed in the same room where the animals were housed, thus their transport to the test cage was not confounded by changes of the surroundings. The animals were weighed weekly on the day at which no barrier tests were performed. The starting weight for females was 180–260 g and for males maximally 230 g to keep the size of their head in a range best suited to the test devices during growth (see below). After completion of the experiment, the animals were used for further experiments such as meningeal CGRP release studies.

### 2.2. Preparation of Animals for Behavioral Tests

To get used to the behavioral set-up and create sufficient motivation for the subsequent experiments, the rats were primed for the attractive drink in advance. In this scheme, they were offered a 10% sucrose solution (in water) on seven consecutive days. The solution (40 mL per animal) was offered by a standard drinking bottle in the housing cage during the same time slot, in which the experiments were later performed, i.e., at 08:00 a.m. 

### 2.3. Test Cage and Recording Device

**Figure 1:**
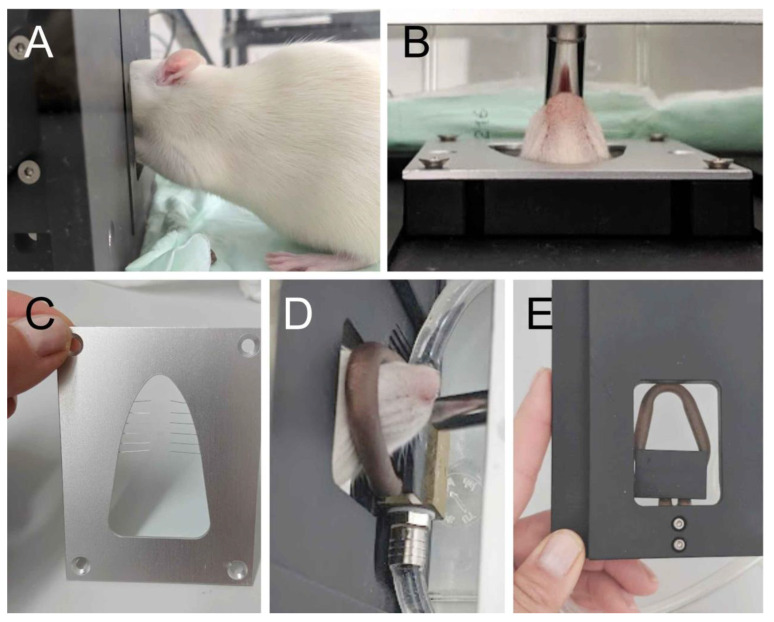
Photographs illustrating details of the experimental setup. The rat sitting in front of the separating wall with the light sensor, has passed the opening with its forehead and is licking the sugar solution from the tube of the drinking bottle (**A**,**B**). A mechanical barrier with 12 bristles (**C**) or a thermal barrier consisting of a U-shaped tube with circulating heated water (**D**,**E**) could be fixed behind the wall.

The behavioral experiments were carried out with the Orofacial Stimulation Test by Ugo Basile (Lugano, Italy; https://ugobasile.com (accessed on 29 March 2023)). The construction consisted of an acrylic glass cage covered by a mesh (48.5 × 27.5 cm, total height 30.5 cm) with a variable recording device, which separated the cage into two parts. In the larger part with approximately 34 × 27.5 cm, the animal could move freely. The other part contained a drinking bottle, which could be adjusted in height, depth, and inclination. An opening in the center of the wall separating the two rooms could be changed in size and shape by inserting different barriers (Figure 1). The animals could approach the outlet of the drinking bottle with their mouth once they passed the opening with their forehead (Figure 1A,B). Mechanical or thermal stimulation barriers could be inserted reducing the size of this opening. A photo sensor (light barrier) was installed directly behind the separating wall in order to detect movements of the rat’s head through the opening. It was important to set the holder for the drinking bottle (filled with 100 mL 10% sucrose solution) correctly so that the light barrier was only interrupted when the animal approached the opening of the bottle to reach the sugar solution. The system automatically recorded the number of drinking trials (approaches) and the duration of intervals when the light barrier was inactivated (finally calculated as the average drinking time per trial and total drinking time). After the experimental period of 15 min had elapsed, the volume of ingested sucrose solution during the test period was determined by measuring the rest of solution in the drinking bottle.

### 2.4. Mechanical and Thermal Stimulation Device

The mechanical barrier that was added to the opening consisted of 12 thin bristles made of flexible spring steel wire (0.09 mm in diameter) that reached at a length of 1.6 cm into the opening (Figure 1C). The barrier constricted the opening and left a circular gap of 0.6 cm free of bristles. When the animal approached the drinking bottle, the facial area between the nose and the eyes was touched by the bristles. For thermal stimulation, the barrier consisted of a tube in the form of a rounded triangular loop (adapted to the contours of the rat’s head) filled with circulating water at a constant flow, which was heated by a water bath and held at a constant temperature of 50 °C (Figure 1E). The opening of the drinking bottle could be reached by the animal while its cheeks and forehead came into contact with the hot tube (Figure 1D). 

### 2.5. Test Procedures

Behavioral experiments were performed at 8:00 a.m. Each experiment lasted 20 min in total. The rat was first placed in the test chamber without the attractant sugar solution and left there for 5 min to get accustomed to the new cage. After this phase, the drinking bottle with the sucrose solution was fixed and left for 15 min. As soon as the drinking bottle was correctly positioned, the test time was started and the light barrier was activated. The experimenter closed the cage with an air-permeable lid. The recording device measured the activity at the light barrier for 15 min. During this time, the rat could freely access the drinking bottle. Food was not offered during the experiment.

### 2.6. Test Sequence

**Figure 2:**
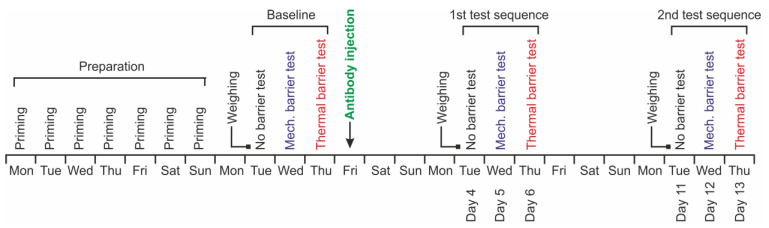
Schematic workflow of the experiments. The first week was used for priming the animals to the attractive source, in the second week baseline measurements without barrier, and with the mechanical and thermal barrier were performed, finished by injection of antibodies (either anti-CGRP antibody fremanezumab or control antibody). In the third and fourth week, the measurements were repeated with the same sequence at days 4–6 and days 11–13 after antibody injection.

All animals in a cage were tested according to the same scheme. The test sequence started after seven days of priming as described above (Figure 2). The tests took place from Tuesday to Thursday for three consecutive weeks. On the first day, each animal was weighed, moved to the test cage, and left there for 5 min to get used to the environment. Then, the drinking bottle filled with 100 mL sugar solution was fixed in place. The program for the test period of 15 min was started. During this period, the cover of the cage remained closed and the animal could move around freely. When this time had elapsed, the cage was opened, the sugar solution removed, and the rat returned to its housing cage. A measuring cylinder was used to read the volume of the sugar solution left and to determine the consumed amount. On the second day of the test sequence, the mechanical barrier was placed into the test cage and the procedure was performed repeating that of the first day. On the third day, the procedure was repeated with the thermal barrier. The entire test sequence in this first week of the experiment was referred to as baseline, while the rats received no antibodies. 

On the day after the third (last) baseline measurement, always on a Friday in this sequence, animals received anti-CGRP antibody or isotype control antibody as described below. On the fourth day after the antibody injection, i.e., on the following Tuesday, the same test sequence as described was repeated, starting without the barrier and repeated with the mechanical and the thermal barrier on the following days. The whole test sequence was repeated at day 11 after the antibody injection. The order of the tested animals was always the same. The experimenter was blinded regarding the treatment of the animals during the whole experiment.

### 2.7. Injection of Antibodies 

The animals were divided into two groups, one group that received isotype control antibody and the other anti-CGRP antibody, fremanezumab (Teva Pharmaceuticals, Redwood City, CA, USA). The rats were transferred from their housing cage in a plastic box and anesthetized around 9 a.m. with an increasing concentration up to 4% isoflurane applied by an evaporator (Forane Vapor 19.3, Dräger AG, Lübeck, Germany). The animals were weighed, individually marked at the tail, and shaved in the neck region. After disinfecting the skin with 70% ethanol, 30 mg/kg anti-CGRP antibody or the same dose of isotype control antibody diluted in saline (10 mg/mL) was subcutaneously injected using a syringe with a 27G needle into the shaved area 2 cm left and right from the midline and 5 cm caudal of the occiput. Examiners performing the injections were blinded to the treatment. The animals were placed back into the housing cage where they recovered from anesthesia within 2–3 min. The animals were inspected two times every following day.

### 2.8. Data Calculation and Statistics

Sequential data (body weight, number of approaches to the drinking source, total drinking time, and drinking volume within 15 min) were calculated for each individual animal. Means were calculated for the whole group and specifically for female and male animals. Statistical analysis was performed with Statistica software (StatSoft, Release 7, Tulsa, OK, USA). Following verification of normal distribution of values, analysis of variance (ANOVA) with repeated measurements was applied, and the factors “antibody” and “sex” were used as independent variables or in combinations. ANOVA was extended by Fisher’s least square difference (LSD) test. The level of significance was set at *p* < 0.05. Data are displayed as the means ± SEM (standard error of the mean). Graphical work was produced using Origin 2017 (https://www.originlab.com (accessed on 29 March 2023)) and CorelDraw X7 (https://coreldraw.com (accessed on 29 March 2023)).

## 3. Results

### 3.1. Allocation of Animals and Tolerability of Treatments 

**Figure 3:**
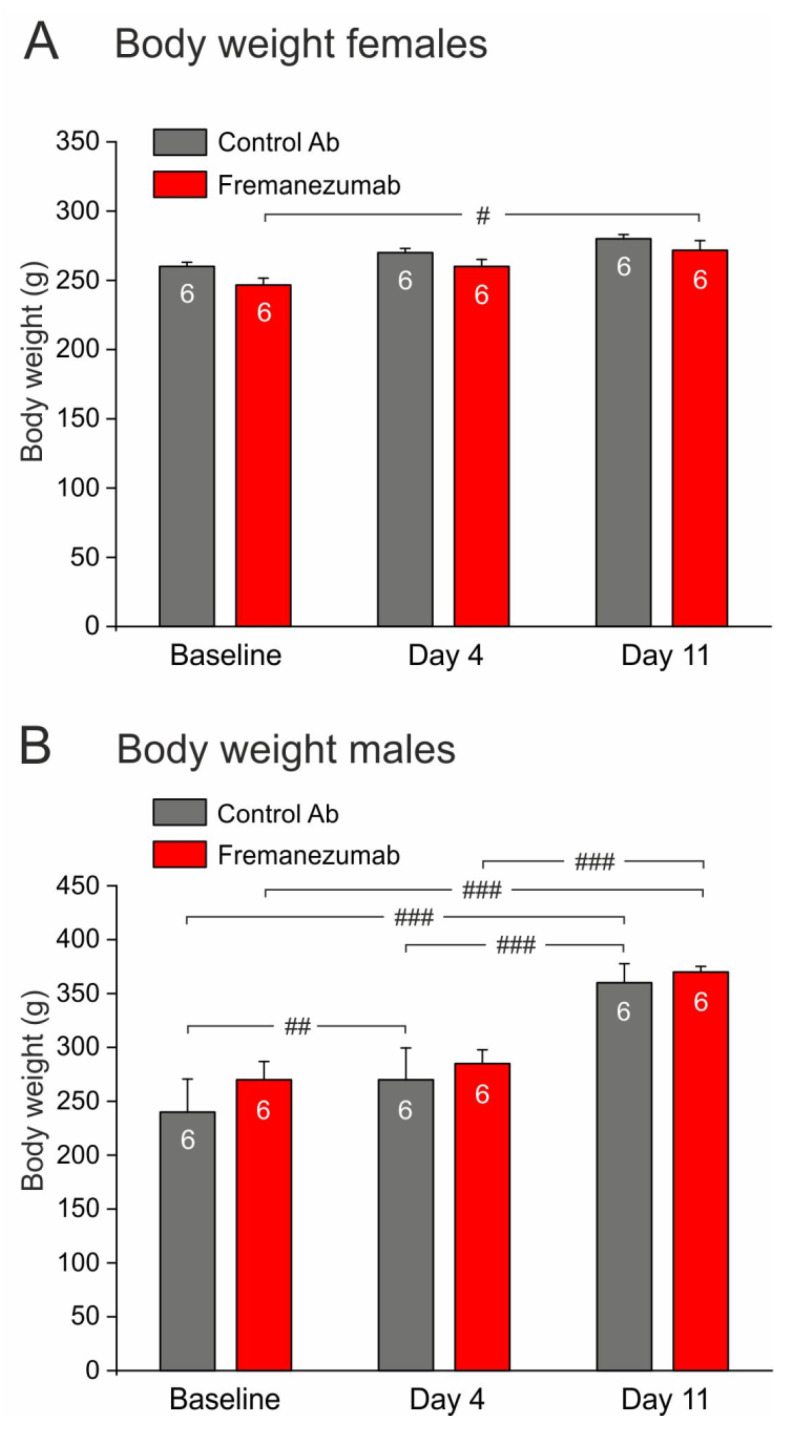
Gain of body weight in animals injected with fremanezumab or control antibody from baseline to the two test sequences at 4 and 11 days. Females (**A**) gained less weight than males (**B**), in which the increase was clearly significant (repeated measures ANOVA with factor antibody, F_2,40_ = 18.9, *p* < 0.0005). There was no significant difference between the animals that received fremanezumab or control antibody in any group at any time of measurement. White digits in the bars represent the number of animals tested. Differences between days: # *p* < 0.05, ## *p* < 0.005, ### *p* < 0.0005 (LSD post hoc test).

Female and male animals (each *n* = 12) were matched according to their age and body weight and, as far as possible, equally allocated into two groups either for control antibody or fremanezumab injection (Figure 2, Baseline). The animals did not take notice of the injection site and did not show any unusual behavior during the following days. During the following two weeks, the animals gained weight, which was more significant in the males than in the females (gain ratio about 4.5:1), but there was no significant difference between the control antibody and fremanezumab groups (Figure 3).

### 3.2. Baseline Experiments

**Figure 4:**
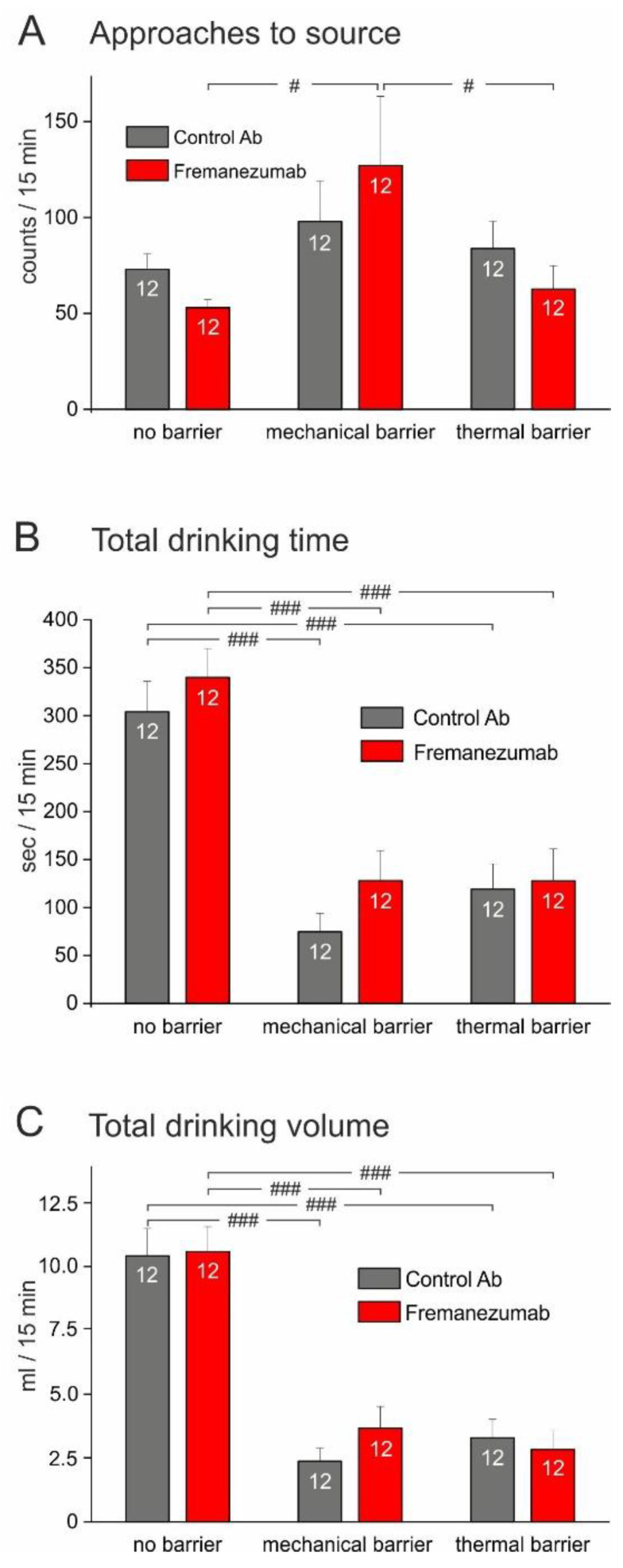
Comparison of baselines for approaches to the source (**A**), drinking time (**B**), and drinking volume (**C**) before the injection of antibodies. Groups designated for the control antibody and fremanezumab, respectively, showed some variance but no significant differences. After inserting the mechanical barrier, the number of trials to approach the source tended to increase (**A**), which was significant in the group designated for fremanezumab (repeated measures ANOVA with factor antibody, F_2,44_ = 3.99, *p* < 0.05 and LSD post hoc test, *p* < 0.05). On the contrary, the mechanical and thermal barriers dramatically reduced both the drinking time (**B**) and drinking volume (**C**) (repeated measures ANOVA, F_2,44_ = 39.17 and 74.03, respectively, *p* < 0.0005). White digits in the bars represent the number of animals tested. Difference between no barrier situation and mechanical and thermal barrier, respectively: # *p* < 0.05, ### *p* < 0.0005 (LSD post hoc test).

In the first week after priming of the animals to the attractive source, i.e., before the injection of antibodies (see Figure 2), we aimed to examine the impact of the test apparatus with the barriers. We counted the number of approaches to the drinking source within 15 min of presentation (Figure 4A), the cumulated time in which the animals stayed at the source inactivating the light barrier (Figure 4B), and the consumed drinking volume within this time (Figure 4C). There was no significant difference between the cohorts of animals designated for injection with control antibody or fremanezumab, respectively, regarding the above measurements without, with the mechanical, and with the thermal barrier (Figure 4A–C). However, after insertion of the mechanical barrier, the number of approaches increased in the fremanezumab group (Figure 4A), while both the drinking time and consumed volume decreased dramatically in both groups when the mechanical or the thermal barrier was inserted (Figure 4B,C).

### 3.3. Experiments after Antibody Injection 

**Figure 5:**
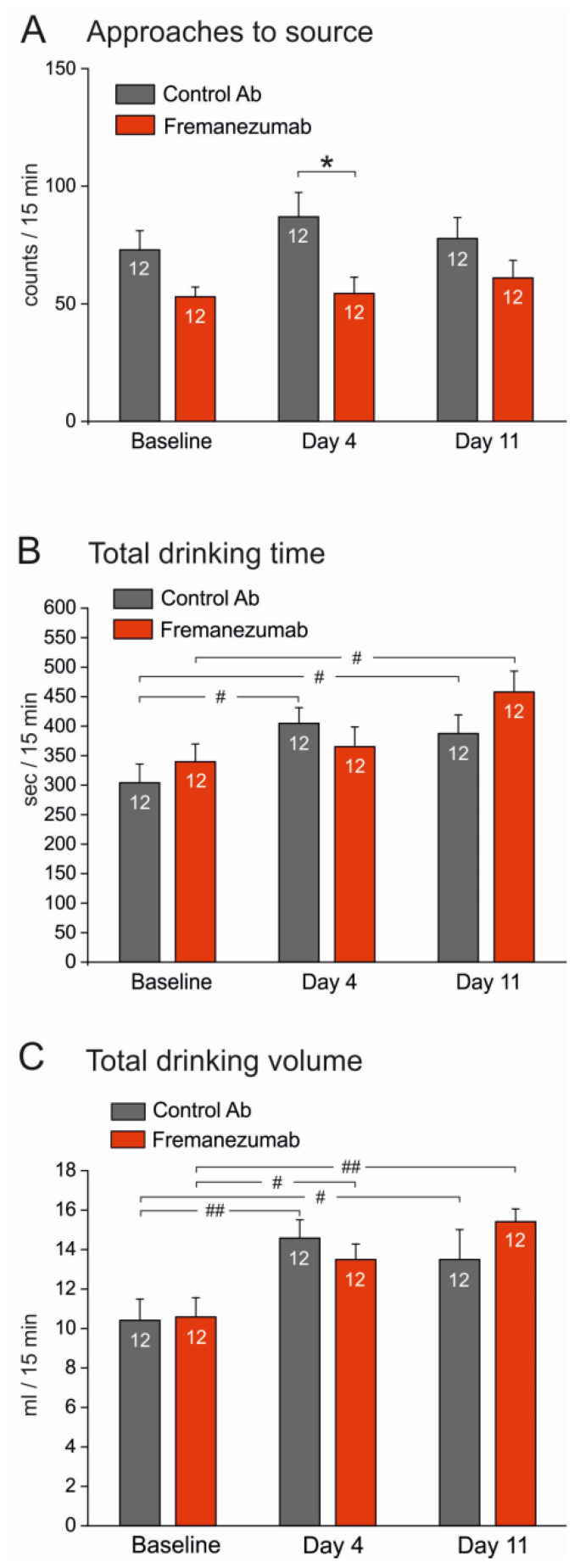
Tests without the barrier before (baseline) and at days 4 and 11 after antibody injection (1st and 2nd test sequence). (**A**) Animals injected with the control antibody tended to approach the drinking source more frequently than animals injected with fremanezumab (repeated measures ANOVA with factor antibody, F_2,44_ = 6.52, *p* < 0.05), which was significant at day 4 after antibody injection (LSD post hoc test, *p* < 0.05). (**B**) The cumulated time, during which the animals stayed at the source to drink, increased at days 4 and 11 compared to baseline, independent of the type of antibody injected (repeated measures ANOVA with factor antibody, F_2,44_ = 8.90, *p* < 0.005). (**C**) Similarly, the cumulated volume consumed by the animals increased at days 4 and 11 (repeated measures ANOVA, F_2,44_ = 17.17, *p* < 0.0005). White digits in bars represent the number of animals tested. Difference between antibodies: * *p* < 0.05; difference in the course of repeated tests: # *p* < 0.05; ## *p* < 0.005 (LSD post hoc tests).

**Figure 6:**
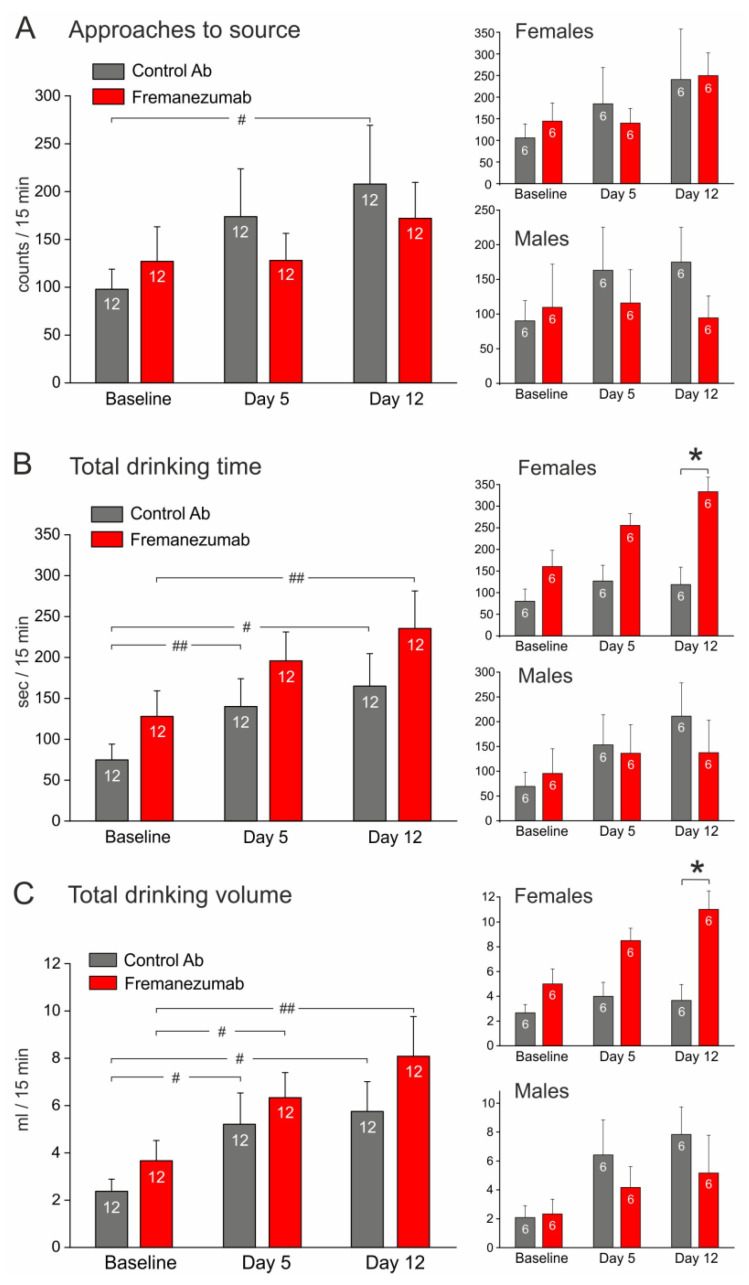
Tests with the mechanical barrier before (baseline) and at days 5 and 12 (1st and 2nd test sequence) after antibody injection. Compared to baseline, the animals tended to approach the drinking source more often (**A**), they stayed longer at the source (**B**), and drank more (**C**), independently of the type of injected antibody (repeated measures ANOVA with factor antibody, F_2,44_ = 8.90, *p* < 0.005 for B and F_2,44_ = 10.44, *p* < 0.0005.81 for (**C**)). However, ANOVA with the combination of factors antibody and sex indicated significant differences (F_1,20_ = 7.28; *p* < 0.05); the LSD post hoc test showed significant differences (*p* < 0.05) in the drinking time and volume exclusively in the female groups at day 12 ((**B**,**C**), right insets). White digits in bars represent the number of animals tested. Difference between antibodies: * *p* < 0.05; difference in the course of repeated tests: # *p* < 0.05; ## *p* < 0.005 (LSD post hoc test).

**Figure 7:**
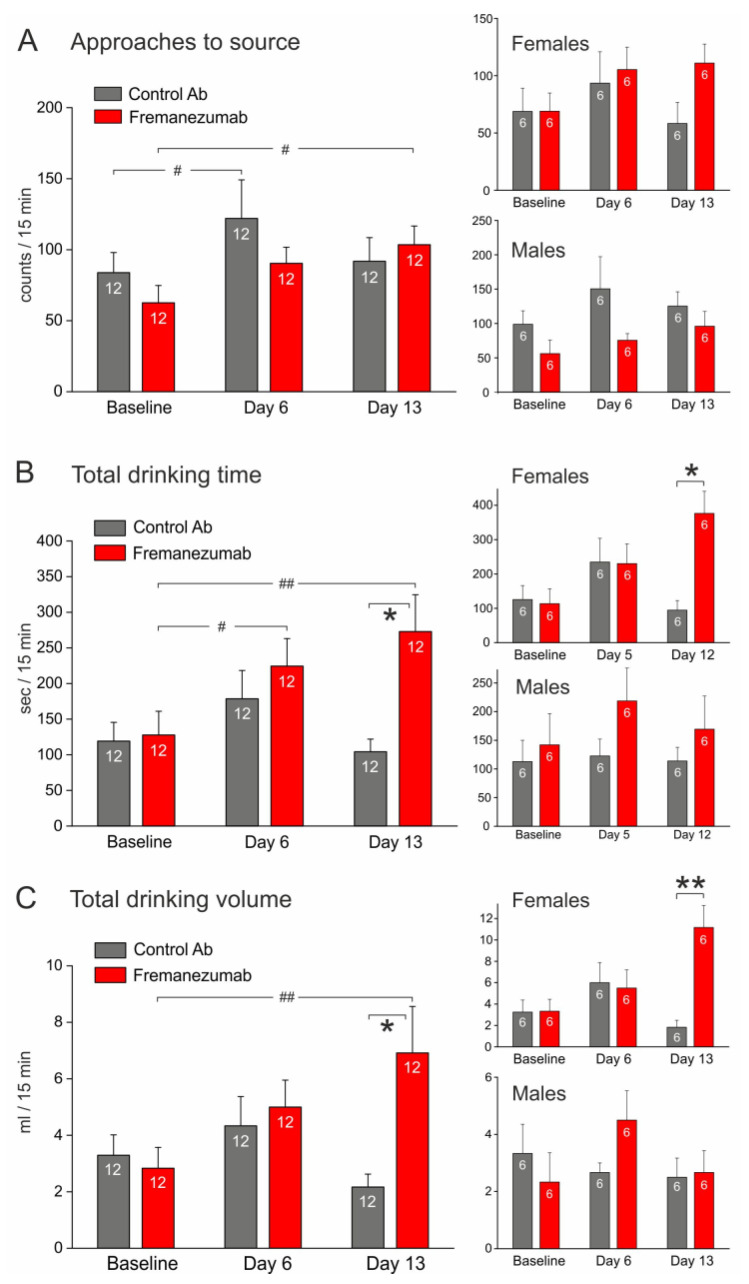
Tests with the thermal barrier before (baseline) and at days 6 and 13 (1st and 2nd test sequence) after antibody injection. At days 6 and 13, the animals partly approached the drinking source more often (**A**) and stayed longer at the source (**B**) compared to baseline, independently of the type of injected antibody (repeated measures ANOVA with factor antibody, F_2,44_ = 3.92, *p* < 0.05 for A and F_2,44_ = 3.60, *p* < 0.05 for B; F_2,44_ = 10.44, *p* < 0.0005.81 for (**C**)). At day 12, the drinking time (**B**) and the consumed volume (**C**) were significantly higher in animals injected with fremanezumab compared to the control antibody (repeated measures ANOVA with combined factors repetition and antibody extended by the LSD post hoc test, F_2,44_ = 45.10, *p* < 0.05). The separated analysis of the sexes showed that this was particularly due to the female animals, which drank longer and significantly more when they were injected with fremanezumab ((**B**,**C**), right insets). White digits in bars represent the number of animals tested. Difference between antibodies: * *p* < 0.05, ** *p* < 0.005; difference in the course of repeated tests: # *p* < 0.05; ## *p* < 0.005 (LSD post hoc test).

In the second and third week after priming (first and second test sequence), i.e., days 4–6 and days 11–13 after antibody injection, the three parameters, number of approaches, cumulated time at the drinking source and consumed solution, were tested again (Figure 5, Figure 6 and Figure 7). The aim was to compare these parameters after injection of the tested antibodies at the first and second test sequence with the baseline data. 

#### 3.3.1. Experiments without Barrier (Figure 5)

The number of approaches to the drinking source tended to decrease after fremanezumab treatment compared to the control antibody in the baseline experiments; the difference was significant in the first test sequence (day 4 after antibody injection) but there was no significant difference between the first test (baseline) and days 4 and 11 when the same groups of animals were compared (Figure 5A). The drinking time and volume increased in the course from the baseline measurements to the first and second test sequence, which was especially significant regarding the drinking volume, most likely as a compensatory mechanism for the increasing body weight (Figure 5B,C). However, there was no difference between the control antibody and fremanezumab at any of the test sequences. A repeated measures ANOVA with the factors “antibody” and “sex” indicated no significant sex difference. 

#### 3.3.2. Experiments with Mechanical Barrier (Figure 6)

The number of approaches to the source tended to increase (Figure 6A), while the drinking time and drinking volume clearly increased in the course from the baseline to the first and second test sequence both in the control antibody and the fremanezumab groups (Figure 6B,C), however, there was no significant difference between the antibodies at same days. At day 12, the drinking time and the consumed volume tended to increase in animals injected with fremanezumab compared to the control antibody. The separated analysis of sexes showed that this was solely due to female animals, which drank longer and significantly more when they were injected with fremanezumab (Figure 6B,C, right insets). 

#### 3.3.3. Experiments with Thermal Barrier (Figure 7)

The number of approaches to the source, drinking time, and volume partly increased in the course of the experiments (Figure 7A–C). A repeated measures ANOVA indicated significant differences in the course of the experiments between antibodies and sexes, the post hoc test showed significance in drinking time and volume between the control antibody and fremanezumab at day 13 (Figure 7B,C). Therefore, the analysis was repeated with the factor “sex”, where ANOVA extended by the LSD test showed that the differences were specifically based on the female groups (Figure 7B,C, right insets). 

## 4. Discussion

To our knowledge, this is the first study using a semiautomatic test system to determine facial mechanical and thermal sensitivity in rodents after treatment with an anti-CGRP monoclonal antibody. Rats of both sexes were primed to use a pleasant-tasting drinking source, which in the experimental condition they could only approach when they touched an unpleasant mechanical or heat barrier with their face. At days 12 and 13 after treatment with anti-CGRP antibody, female, but not male, animals stayed significantly longer at the source and consumed more volume compared to animals treated with the control antibody. We conclude that animals treated with anti-CGRP antibody, in particular females, show a higher tolerance towards unpleasant facial mechanical and heat stimuli. Our data suggest that facial sensitivity is partly dependent on CGRP levels which are lowered by anti-CGRP antibodies. 

### 4.1. Test Device

The semiautomatic device deployed for this study is new and needed mechanical adjustment for its proper use. In particular, the mechanical barrier with the steel bristles had to be modified and adapted in size for the head of the animals, which required several trials in preliminary experiments. In previous studies, facial sensitivity was most frequently tested by determining the withdrawal threshold with hand-held calibrated von Frey filaments, which requires fixing the animals and repetitive testing at exactly the same site [19,20,21,22]. Different to these, in our test device the animals were free to contact the unpleasant barriers and could do this in their individual way, only attracted by the sweet drink. In the course of the test sequence, no differences between the tested antibodies became apparent as long as no barriers were used. This suggests that the animals did not undergo any fundamental change in behavior as a result of antibody application. The mechanical and thermal barriers proved to be effective in terms of nociceptive stimulation of the periorbital area, as was evident from the dramatic drop in drinking time and volume when the barriers were fixed. As a confounding factor, the number of approaches to the source and the duration of drinking was possibly also influenced by psychological factors such as motivation, attendance, and alertness of the animals. The device used in our study is reminiscent of an operant test system previously developed to determine mechanical and thermal orofacial sensitivity [23,24]. Similar to this device, a thermal stimulation paradigm was added in our experiments, which has rarely been used so far regarding facial sensitivity in a behavioral context [25,26,27]. The observation that heat stimulation yielded very similar results to the mechanical barrier may argue for an involvement of polymodal C-nociceptors in the CGRP-dependent modulation of facial sensitivity [28]. 

### 4.2. Proposed Mechanisms of Anti-CGRP Antibody Effects

Fremanezumab is a humanized monoclonal antibody that specifically targets CGRP and antagonizes CGRP-induced cAMP signaling at the canonical human CGRP receptor [29]. This antibody has been shown to also inhibit the vasodilatory effect of rat CGRP in rat cranial arteries [30] and the activity of rat meningeal afferents [7]. In our previous study, fremanezumab reduced CGRP release from rat trigeminal tissues as well as increasing meningeal blood flow evoked by stimulation with the TRPV1 receptor agonist capsaicin [31]. Therefore, although human and rat α- and β-CGRPs differ in four amino acids [32], fremanezumab binds rat CGRP and is suitable for testing CGRP-mediated effects in this species. The results of the present study suggest that the anti-CGRP antibody, fremanezumab, decreased periorbital sensitivity to noxious mechanical and heat stimuli and hence facial sensitivity may be influenced by CGRP. This result was unexpected, since a decrease in the facial mechanical threshold upon inhibition of CGRP signalling has so far only been reported in experiments in which the trigeminal system was sensitized, for example by repetitive electrical stimulation [33] or cortical spreading depolarization [27]. Injection of CGRP into the trigeminal ganglion has also been reported to cause periorbital mechanical allodynia attenuated by sumatriptan [34]. 

We speculate that the decrease in facial sensitivity following treatment with anti-CGRP antibodies is not merely a function of peripheral CGRP actions but may also include central effects. In a mouse model, CGRP administration produced facial grimace indicative of spontaneous pain, which was blocked by pre-administration of a monoclonal anti-CGRP antibody [13]. Avoidance of unpleasant or painful stimuli is a complex behavior depending on several factors such as motivation and tolerability. The observation that 12–13 days passed until the effect of fremanezumab was apparent may be interpreted with such a behavioral adjustment. In a broader clinical context, these observations are reminiscent of the real life experiences with monoclonal antibodies in the treatment of chronic migraines, which have been found not only to decrease the frequency and duration of migraine attacks but also the severity reported by the patients [35,36]. 

### 4.3. Sex Differences

Although the animals seemed to tolerate the mechanical and thermal barrier longer after treatment with fremanezumab in all groups tested, the differences to the control antibody were statistically significant only in female animals. We did not test and correct for the oestrus cycle of the females, which would not have been compatible with the fixed test sequence. Sex differences in facial sensitivity of rodents are known from several reports. In rat models of headache, female animals showed more frequent headache-like behavior [37]. Following meningeal afferent sensitization through application of inflammatory mediators onto the dura mater in rats, females but not males showed increased withdrawal responses to mechanical test stimuli in the periorbital region [38]. Similarly, application of small doses of CGRP onto the dura mater caused periorbital hypersensitivity only in female rats, and particular sex-specific sensitivity to CGRP was caused by the application of interleukin-6 or the NO-donor sodium nitroprusside [17]. In a model of post-traumatic headache, female rats developed pericranial mechanical hyperalgesia that lasted longer than in males and showed elevated CGRP levels in the peripheral blood [39]. Thus, we can conclude that female rats are more sensitive to CGRP, which may be the reason why anti-CGRP antibody treatment may be more efficient in female rats. 

The mechanisms underlying the higher sensitivity to CGRP of female rats are not clear. We assume that sex hormones such as estrogen with a higher expression in the female rat body independent of the estrus state contribute to sensitizing the recently proposed CGRP-NO-TRPA1 signalling cascade, inducing facial hypersensitivity [40]. In this cascade CGRP, released mainly from trigeminal C-afferents, is proposed to activate CGRP receptors on adjacent Schwann cells (glial cells of Remak bundles). This induces the expression of nitric oxide (NO) synthase to produce more NO, which in turn acts on TRPA1 receptor channels of adjacent Aδ-afferents causing afferent sensitization. This hypothetical cascade is based on previously published experiments in which this group has demonstrated that subcutaneous injection of CGRP and other migraine-provoking substances cause periorbital mechanical hypersensitivity in mice [15]. Regarding sex differences, it has formerly been shown that inhibition of the vascular NO synthase has different effects in pre- versus post-menopausal women and men in that vasoconstriction was selectively higher in the pre-menopausal state [41], indirectly indicating that sex hormones such as estrogen may contribute to an increase in endogenous NO production. Regarding facial hypersensitivity as an animal model of migraine, it would be interesting to examine whether female migraineurs, particularly in the pre-menopause, may benefit more from a treatment with monoclonal anti-CGRP antibodies than male migraineurs. Overall, these results fit to the generally higher prevalence of migraine in women [42].

### 4.4. Clinical Relevance and Limitations

Migraine patients frequently show facial allodynia and are also hypersensitive in other body regions [43,44]. To our knowledge, changes in cutaneous sensitivity have not been systematically monitored in patients treated with fremanezumab. Following repetitive treatment with galcanezumab, another anti-CGRP antibody, cutaneous allodynia during migraine attacks has been found to improve, however, without reaching statistical significance after correction for multiple comparison [45]. Similar late responses including the decrease in allodynia to galcanezumab, fremanezumab and the anti-CGRP receptor antibody erenumab have recently been communicated [46]. These data are based on self-reports of patients and did not differentiate between body regions. It would be interesting to test, in addition to facial sensitivity, other regions of the rat’s body with the device used in our experiments.

Thermal hypersensitivity is usually not tested in migraine, and patients usually do not report thermal hypersensitivity. Thermal pain thresholds have been shown to be decreased immediately prior to migraine attacks [47]. Derived from the present data, we assume that thermal hypersensitivity in the face of migraineurs may be reduced after treatment with anti-CGRP antibodies. Developing a standard test for the evaluation of mechanical and thermal sensitivity in migraineurs would possibly be very useful for monitoring the effect of treatments targeting CGRP or its receptors. 

## 5. Conclusions

In conclusion, the current experiments together with our previous results about CGRP release [31], and earlier data about reduced trigeminal activity [7] and prevention of periorbital allodynia [48] following pre-treatment with the anti-CGRP antibody fremanezumab strongly argue for a role of CGRP in the development of facial sensitivity and hyperalgesia, at least in female animals. The experiments confirm this rodent model based on semi-automated recording of facial sensitivity is useful for preclinical research on headache therapies targeting CGRP signalling. 

## Data Availability

Not applicable.
